# Uncovering fungal community composition in natural habitat of *Ophiocordyceps sinensis* using high-throughput sequencing and culture-dependent approaches

**DOI:** 10.1186/s12866-020-01994-2

**Published:** 2020-11-02

**Authors:** Chuan-Bo Zhang, Chao-Hui Ren, Yan-Li Wang, Qi-Qi Wang, Yun-Sheng Wang, Qing-Bei Weng

**Affiliations:** School of Life Sciences, Guizhou Normal University, Huaxi University Town, Gui’an New District, Guiyang, 550025 China

**Keywords:** Fungal communities, *Ophiocordyceps sinensis*; External mycelial cortices, Soil microhabitats, Culturable fungi

## Abstract

**Background:**

The fungal communities inhabiting natural *Ophiocordyceps sinensis* play critical ecological roles in alpine meadow ecosystem, contribute to infect host insect, influence the occurrence of *O. sinensis*, and are repertoire of potential novel metabolites discovery. However, a comprehensive understanding of fungal communities of *O. sinensis* remain elusive. Therefore, the present study aimed to unravel fungal communities of natural *O. sinensis* using combination of high-throughput sequencing and culture-dependent approaches.

**Results:**

A total of 280,519 high-quality sequences, belonging to 5 fungal phyla, 15 classes, 41 orders, 79 families, 112 genera, and 352 putative operational taxonomic units (OTUs) were obtained from natural *O. sinensis* using high-throughput sequencing. Among of which, 43 genera were identified in external mycelial cortices, *Ophiocordyceps*, *Sebacinia* and *Archaeorhizomyces* were predominant genera with the abundance of 95.86, 1.14, 0.85%, respectively. A total of 66 genera were identified from soil microhabitat, *Inocybe*, *Archaeorhizomyces*, unclassified *Thelephoraceae*, *Tomentella*, *Thelephora*, *Sebacina*, unclassified *Ascomycota* and unclassified fungi were predominant genera with an average abundance of 53.32, 8.69, 8.12, 8.12, 7.21, 4.6, 3.08 and 3.05%, respectively. The fungal communities in external mycelial cortices were significantly distinct from soil microhabitat. Meanwhile, seven types of culture media were used to isolate culturable fungi at 16 °C, resulted in 77 fungal strains identified by rDNA ITS sequence analysis, belonging to 33 genera, including *Ophiocordyceps*, *Trichoderma*, *Cytospora*, *Truncatella*, *Dactylonectria*, *Isaria*, *Cephalosporium*, *Fusarium*, *Cosmospora* and *Paecilomyces*, etc.*.* Among all culturable fungi, *Mortierella* and *Trichoderma* were predominant genera.

**Conclusions:**

The significantly differences and overlap in fungal community structure between two approaches highlight that the integration of high-throughput sequencing and culture-dependent approaches would generate more information. Our result reveal a comprehensive understanding of fungal community structure of natural *O. sinensis*, provide new insight into *O. sinensis* associated fungi, and support that microbiota of natural *O. sinensis* is an untapped source for novel bioactive metabolites discovery.

## Background

The caterpillar fungus *Ophiocordyceps sinensis* parasitizes the larvae of *Thitarodes* that belongs to family Hepialidae (Lepidoptera) form the fungus-caterpillar complex (Chinese *Cordyceps*) [1]. *O. sinensis* mainly inhabits the alpine meadow ecosystem of Qinghai–Tibetan Plateau, Sichuan, Yunnan and Gansu Provinces in China, also distributes in certain areas of Bhutan, Nepal and India [2]. As the well-known traditional Chinese medicinal herb, *O. sinensis* has been widely used for centuries in Asia, exhibit beneficial properties and remedy for a variety of chronic disorders, including asthma, cancer, sexual dysfunction, obesity, type 2 diabetes and fatigue, etc., presumably, by modulating the composition of gut microbiota [3–6]. *O. sinensis* harbour a variety of bioactive metabolites, e.g., cordycepin, cordycepic acid, polysaccharides, ergosterol, mannitol and macrolides, etc.. It may be considered as key factors in the coevolution of *O. sinensis* and its host, coevolution of symbiotic and associated microbiota. The huge world market demand and price of *O. sinensis* continue to increase because of its pharmacological application, obligate parasitism and special dependence on ecological environments, which led to overexploitation, severely endangering numerous natural populations of *O. sinensis* towards its extinction and damage of its habitats. Artificial cultivation of *O. sinensis* has been successful, but commercial cultivation is difficult to perform because the cryptic factors triggering the development and maturation of *O. sinensis* remains obscure [7]. Currently, the soil originated from habitats of natural *O. sinensis* is indispensable in later stages of artificial cultivation. Therefore, illuminating mysterious drive factor in the occurrence and maturation of *O. sinensis*, enhancing the yield and discovery of its alternative substitutes, have already become hot topics.

Generally, *O. sinensis* is exclusively endemic to alpine meadow ecosystem of the Qinghai-Tibetan Plateau and its adjacent high-altitude area in China, which is characterized by low soil temperature, fragile plant community structure and extremely sensitive to climate change. Natural *O. sinensis* and its surrounding soil construct a microecosystem, which represent the reservoir of microbial species. Uncovering the fungal community structure of natural *O. sinensis* are significant in understanding the occurrence of *O. sinensis* and artificial cultivation. Recently, a wide variety of fungal species have been discovered from natural *O. sinensis* using culture-dependent approach, including *O. sinensis*, *Chrysosporium sinense*, *Paecilomyces hepiali*, *Tolypocladium sinense* and *Paecilomyces sinensis*, etc., all of which are excellent potential sources of novel bioactive metabolites for drug discovery [8, 9]. Except the caterpillar fungus *O. sinensis*, all the others are symbiotic or associated fungi [10, 11]. Molecular evidence demonstrates the existence of both *O. sinensis* and *P. hepiali* DNA in the caterpillars and fruiting bodies of *O. sinensis.*

Recently, *O. sinensis* is confronted with reduction of output and geographical distribution shrink, which might result from microbial community disturbance in habitat environment by over-exploitation, global warming, heavy grazing and habitat deterioration, etc. [12]. Significantly, a variety of fungal species isolated from natural *O. sinensis* were applied in clinical treatment because of its health benefits. More than 200 novel bioactive metabolites have been isolated from these associated fungi (refered to as *Cordyceps*-colonizing fungi) [13, 14]. Particularly, the mycelia of *P. hepiali* obtained by solid state fermentation or submerged liquid fermentation produce a large repertoire of bioactive metabolite similar to natural *O. sinensis*, also exhibit beneficial properties, including antioxidant, anti-fatigue, anti-aging, anti-diabetic, anti-inflammatory and anti-depressant-like effects [15, 16]. Currently, *P. hepiali* has been widely used as an alternative to natural *O. sinensis*, because of its high productivity and similar health effects. These different fungal species produce similar bioactive metabolites, presumably, due to horizontal transfer of secondary metabolic biosynthetic gene cluster in microbiota of natural *O. sinensis*. Meanwhile, more fungal species originating from natural *O. sinensis* need to be discovered for developing alternative products, and applied in clinical practice.

Natural *O. sinensis* associated microbiota play vital roles in nutrient cycling, establishment of infection cycle of insect hosts, growth and maturation of sclerotia and fruiting body (stroma). Within the last decade, progressive studies have been devoted to isolate and identify fungal species from habitats of *O. sinensis* by culture-dependent approach. A total of 572 isolates have been obtained from different parts of *O. sinensis* (fruiting body, sclerotia and external mycelial cortices) at 25 °C, belonging to 37 fungal genera, Ascomycota, Basidiomycota and Zygomycota were the predominant fungal phyla, which have provided fundamental insights into fungal community of native *O. sinensis* on the Qinghai-Tibetan Plateau [8]. However, only a small fraction of microbial community were culturable using traditional culture dependent approach [17, 18]. Previously, 490 fungal clones were identified from the whole-community DNA using PCR-single-strand conformation polymorphism (PCR-SSCP) method, 266 of these clones were selected for sequencing and assigned to 21 genera [9]. Three main phyla, including Ascomycota, Basidiomycota, Zygomycota and a notable numbers of unclassified fungi, especially, in the sclerotia and external mycelial cortices were detected by high-throughput 454 sequencing technology [19].

Currently, Illumina Miseq high-throughput sequencing has been the most promising approach to explore microbial community composition and dynamic across special habitat, agriculture, traditional solid-state fermentation, deep-sea sediments and Chinese liquor starter, etc., due to its cost-effective, more sequences per sample, especially the capability of less abundant species mining [20–25]. However, the data obtained from samples by this approach were determined by the quality of microbial DNA extracted from samples, PCR amplification efficiency, quality of 16S rRNA or ITS regions library and other uncertain factors, which easily lead to offset or error of microbial diversity. In addition, due to short length of Miseq sequencing, the OTUs could be identified to the genus level.

Recently, *O. sinensis* associated fungi are increasingly attracting the attention as potential health benefits agent. The relationship between *O. sinensis* and its microbiota also represents one of the most evolutionarily ancient examples of symbiosis in ecosystem. The microbial community of unfertilized eggs from *Thitarodes* were analyzed via 16S rRNA and ITS sequencing, revealed that 348 bactetial genera belong to 26 phyla, and 289 fungal genera, mainly including *Aureobasidium*, *Candida* and *Cryptococcus*, etc., belonging to 5 phyla, namely, Ascomycota, Basidiomycota, Chytridiomycota, Glomeromycota and Zygomycota [26]. However, microbial communities of natural *O. sinensis*, ecological functions and metabolic potential remain largely unexplored. Here, the present study aimed to comprehensively uncover fungal communities in external mycelial cortices and soil microhabitat. The samples derived from different sites distributed across Golog Tibetan Autonomous Prefecture of Qinghai-Tibetan Plateau, were investigated using high-throughput sequencing and various types of culture media. These results would provide a comprehensive understanding of complex microbial ecosystem around natural *O. sinensis*, be beneficial to overcome the obstacle in artificial culture of *O. sinensis.*

## Results

### Quality analysis of sequencing data

A total of 280,519 high quality fungal sequences were generated from six samples (external mycelial cortices: JM-1, JM-2, JM-3, soil microhabitat: TY-2, TY-3, TY-4) using Illumina Miseq sequencing. Each sample provided more than 32,568 fungal ITS sequences with an average length of 280.54 bp. The high quality sequence length of the samples from soil microhabitat is 200 ~ 340 bp, the average length is 307.02 bp. The high quality sequence length of the samples from external mycelial cortices is 240 ~ 260 bp, and the average length is 254.06 bp, which is consistent with the prediction based on the primer site (Table 1).

**Table 1:** Quality analysis of sequences from different samples

Sample	Sequences	Bases (bp)	Average Length (bp)
TY2	32,568	10,530,892	323.35
TY 3	37,531	11,194,578	298.28
TY 4	87,493	16,197,280	299.42
JM 1	35,546	9,036,787	254.23
JM 2	42,817	10,862,727	253.70
JM 3	44,564	11,330,766	254.25

### Richness and diversity of fungal community in natural *O. sinensis*

**Fig. 1:**
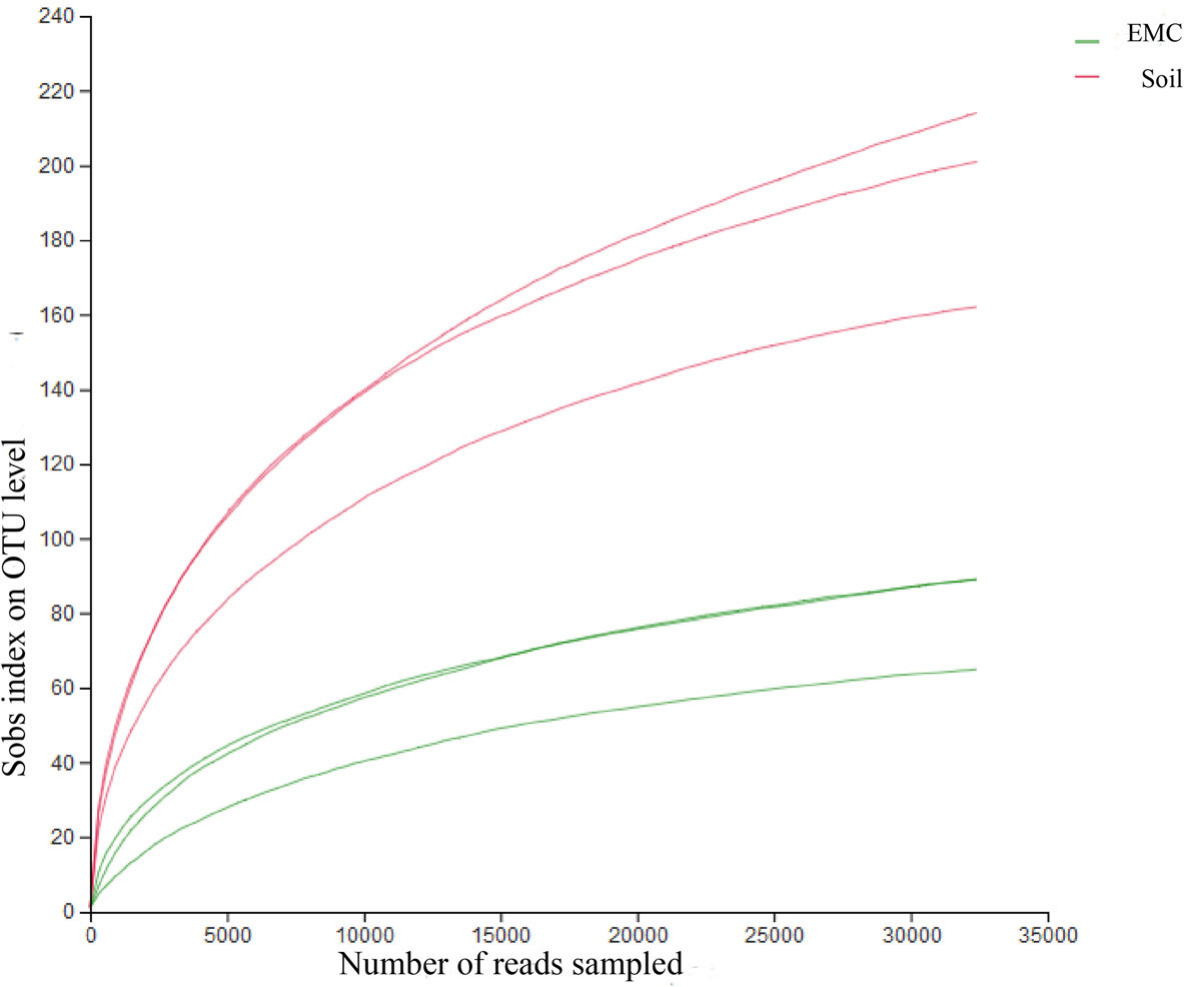
Rarefaction curves of OTUs clustered at < 97% sequence identity for the six samples

In all six samples derived from external mycelial cortices and soil microhabitat, the coverage values close to 100%, and the rarefaction curves were asymptotic, indicating that overwhelming majority of the fungal species were covered (Fig. 1). The total number of ITS reads obtained from all six samples, after filtering chimeric sequences and mismatches, were clustered into 352 OTUs with at 97% similarity in nucleotide identity. One hundred and seventy OTUs were derived from the external mycelial cortices, 291 OTUs were derived from the soil microhabitat, and 109 OTUs were shared.

To better understand the differences among the microbial communities, it is important to calculate the richness and diversity. The species richness of fungal communities can be demonstrated by Chao1 index and ACE index. Simpson index and Shannon index were used to analyze the diversity of fungal community, which demonstrate not only the species richness but the evenness of the species. In natural *O. sinensis*, the patterns of Chao1 and ACE are very similar to the OTUs numbers, the Chao1 and ACE indices from external mycelial cortices are 102.9 ± 23.0, 105.1 ± 21.9, respectively, the Chao1 and ACE indices from soil microhabitat are 241.4 ± 56.0, 248.9 ± 50.4, respectively, which indicate that the external mycelial cortices had lower species richness of the fungal community than soil microhabitat (Table 2). The Shannon and Simpson index of the external mycelial cortices were 0.28, 0.9166, the soil samples were 1.89, 0.3367, respectively. The Shannon index of external mycelial cortices was lower than soil microhabitat, in contrast, the Simpson index of external mycelial cortices was higher than soil microhabitat. The results indicate that taxonomic diversity of the fungal community in soil microhabitat are higher than external mycelial cortices.

**Table 2:** Richness and diversity of the fungal community from samples at 97% similarity

Sample	OTU	ACE index	Chao1 index	Shannon index	Simpson index
Soil	291	248.9 ± 50.4^a^	241.4 ± 56.0^a^	1.89 ± 0.55^a^	0.3367 ± 0.2104^a^
EMC	170	105.1 ± 21.9^b^	102.9 ± 23.0^b^	0.28 ± 0.14^b^	0.9166 ± 0.0450^b^

Values are means±standard deviation, different letters (a,b) indicate significantly different (*p* < 0.05)

### Fungal community structure analysis of *O. sinensis* using high-throughput sequencing

**Fig. 2:**
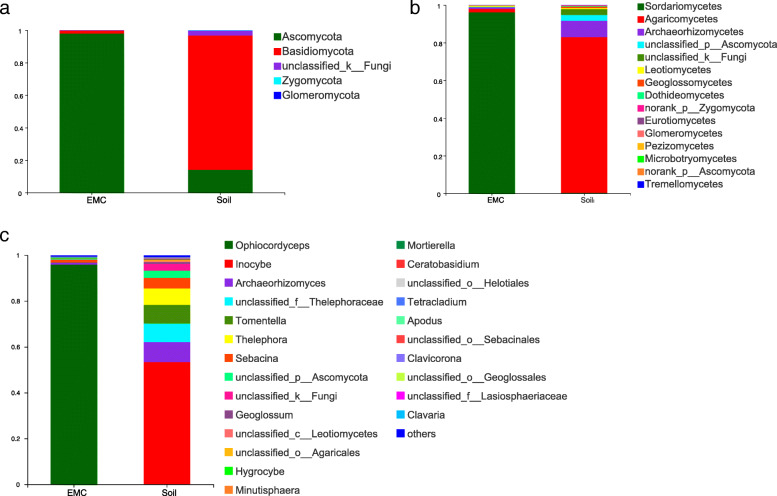
Taxonomic profiles of natural *O.sinensis* associated fungal communities at different taxonomic levels. **a** phylum; **b** class; **c** genus

The fungal community structure of *O. sinensis* were evaluated at the phylum, class and genus levels. All high-quality sequences generated from external mycelial cortices and soil microhabitat belong to 5 fungal phyla, 15 Classes, 41 orders, 79 families, 112 genera, 352 OTUs. A total of 4 fungal phyla were identified in external mycelial cortices samples, including Ascomycota, Basidiomycota, Zygomycota and unclassified fungi, the average abundance were 98.02, 1.79, 0.07, 0.12%, respectively. Ascomycota was the predominant fungus in external mycelial cortices samples of natural *O. sinensis.* A total of 5 fungal phyla were identified in soil microhabitat samples, including Basidiomycota, Ascomycota, Zygomycota, Glomeromycota and unclassified fungi, the abundance were 82.66, 14.06, 0.18, 0.05 and 3.05%, respectively (Fig. 2a). Significantly, Basidiomycota and Ascomycotawas were the predominant fungi in soil microhabitat, and Glomeromycota was not detected in external mycelial cortices. The proportions of Ascomycota in the external mycelial cortices of *O. sinensis* were significantly higher than soil microhabitat.

The composition of fungal community were further analyzed at the genus level. Among 5 fungal phyla, a total of 112 genera were identified across all investigated samples. In total, 43 genera were discovered in external mycelial cortices, *Ophiocordyceps*, *Sebacina* and *Archaeorhizomyces* were predominant genera accounted for 95.86, 1.14, 0.85%, and the genus *Ophiocordyceps* was overwhelmingly dominant in external mycelial cortices. A total of 66 genera were discovered in soil microhabitat, *Inocybe*, *Archaeorhizomyces*, unclassified *Thelephoraceae*, *Tomentella*, *Thelephora*, *Sebacina*, unclassified *Ascomycota*, unclassified fungi were dominant genera with an average abundance of 53.32, 8.69, 8.12, 8.12, 7.21, 4.6, 3.08 and 3.05%, respectively. In total, 34 genera were shared, among of which, *Sebacina*, *Archaeorhizomyces*, *Apodus*, *Tetracladium*, *Mortierella* and *Cistella* had higher abundance in external mycelial cortices, accounted with 1.14, 0.85, 0.18, 0.17, 0.07, 0.04%, respectively. However, *Inocybe*, *Archaeorhizomyces*, *Tomentella*, *Thelephora*, *Sebacina* and *Geoglossum* had higher abundance in soil microhabitat, accounted with 53.32, 8.69, 8.12, 7.21, 4.61, 0.74%, respectively. The average abundance of other genera are lower than 0.01%.

In addition, a total of 9 genera, namely, *Botrytis*, *Cladosporium*, *Coniochaeta*, *Hygrocybe*, *Lachnum*, *Laetisaria*, *Leucosporidiella*, *Ophiocordyceps* and *Trichoderma* were unique to external mycelial cortices. A total of 32 genera were unique to soil microhabitat samples, *Acremonium*, *Alternaria*, *Ambispora*, *Aspergillus*, *Basidiobolus*, *Cercophora*, *Clavaria*, *Clavulinopsis*, *Cotylidia*, *Cryptococcus*, *Elaphomyces*, *Elasticomyces*, *Entoloma*, *Glarea*, *Gloiocephala*, *Humicola*, *Hymenula*, *Lecythophora*, *Leptosphaeria*, *Minutisphaera*, *Monographella*, *Mrakia*, *Mycena*, *Neopeckia*, *Ophiosphaerella*, *Penicillium*, *Pluteus*, *Porotheleum*, *Rhodotorula*, *Russula*, *Scutellinia* and *Tarzetta.* Among of which, *Minutisphaera* and *Scutellinia* have higher abundance of 0.29, 0.02%, respectively, the others with an average abundance less than 0.01% (Fig. 2c).

### Comparison of fungal communities from different samples in natural *O. sinensis*

**Fig. 3:**
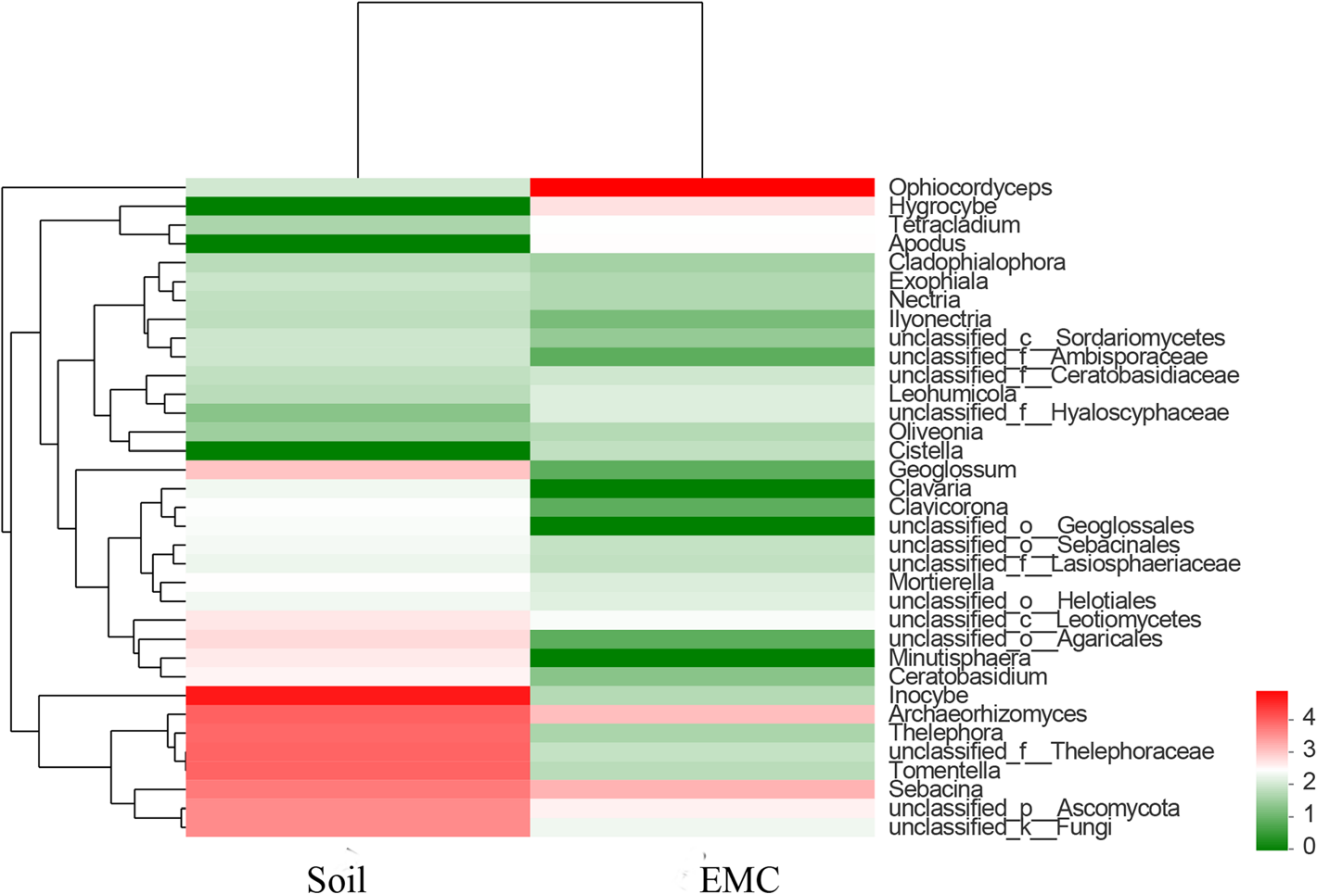
Relative abundance of fungal community proportions at the genus level in external mycelial cortices and soil microhabitat samples. The heatmap illustrates the abundance of the top 35 genera, different colour indicates difference relative abundance of the taxa in all samples, the scale bar shows the variation range of the normalized abundance of the genera (The relative abundance of fungal community from high to low is represented by red and green)

**Fig. 4:**
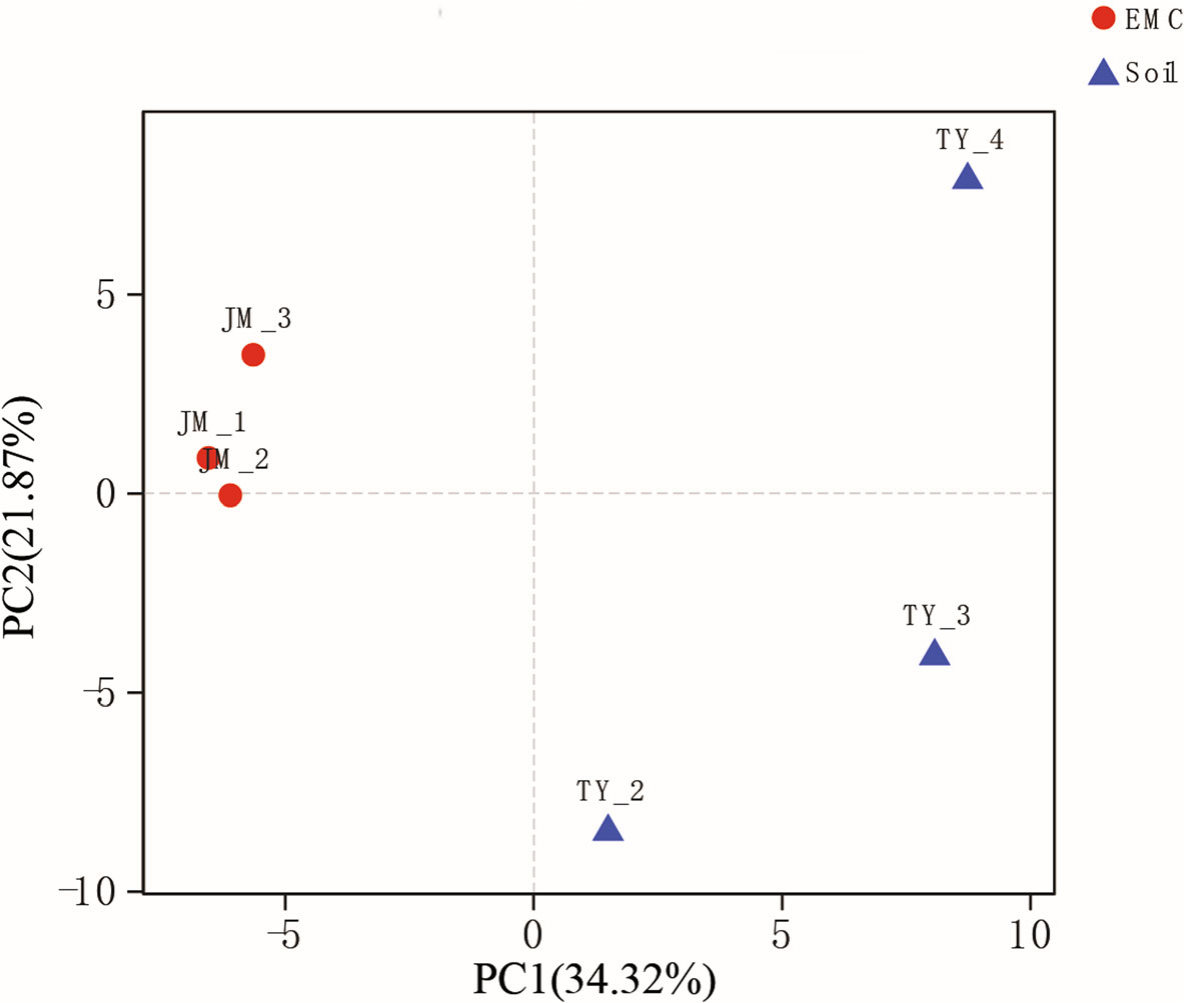
Principal coordinates analysis of fungal communities in samples of external mycelial cortices and soil microhabitat. The OTU matrix used in the analyses was clustered at the 97% similarity and the principal coordinate’s analysis was based on Weighted UniFrac distances. The solid points in abbreviations of the sample name indicate the samples distributed in the ordination

**Fig. 5:**
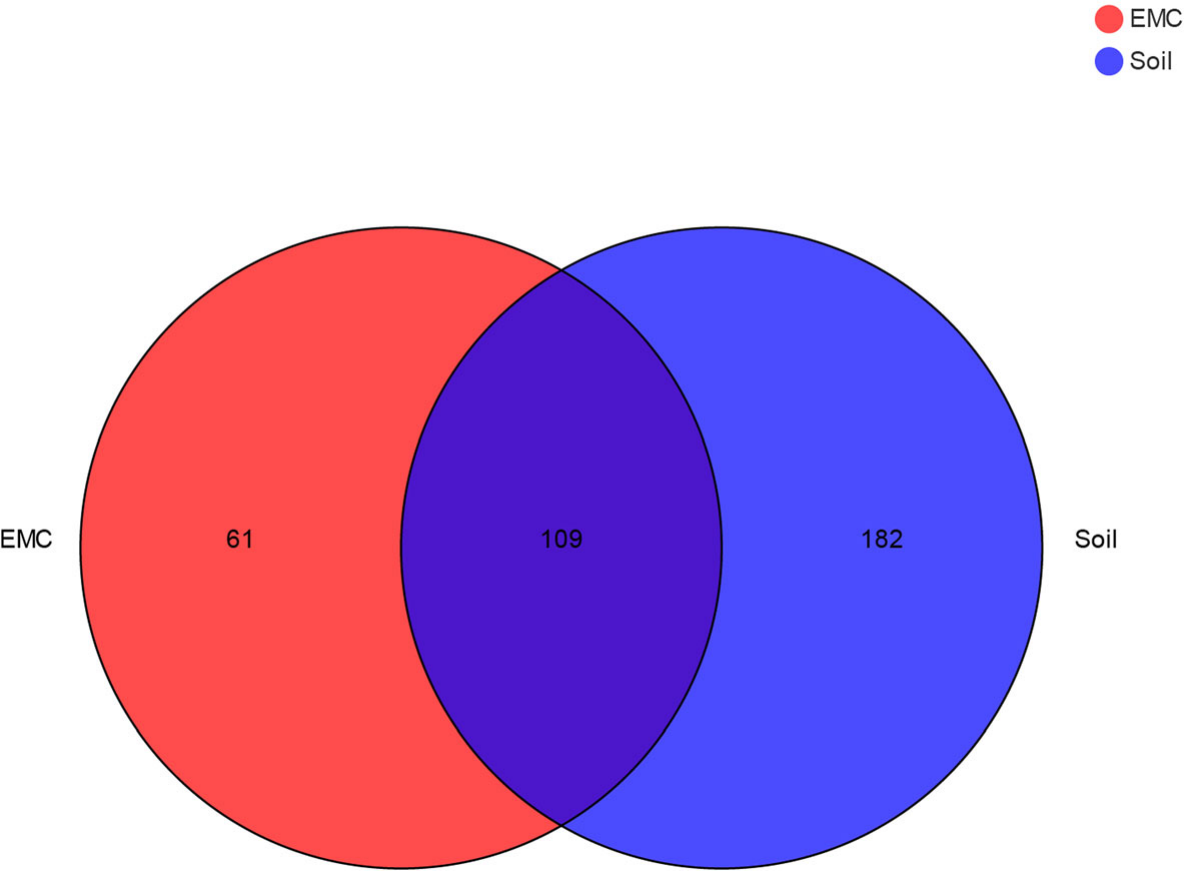
Venn diagram for fungal communities. Venn diagram showing the number of shared and unique OTUs (≥97% similarity) among the external mycelial cortices and soil microhabitat

**Fig. 6:**
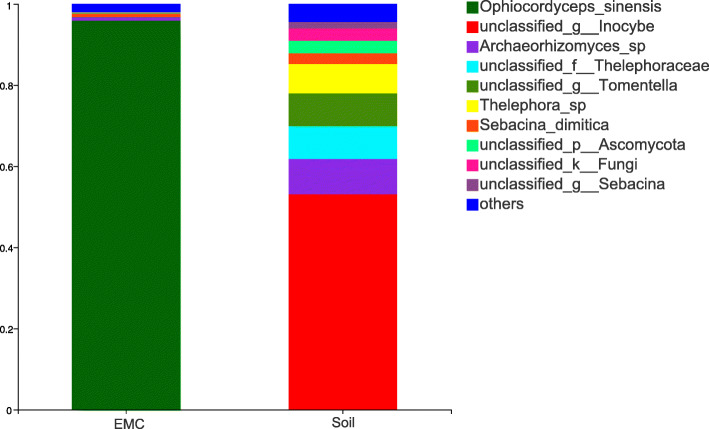
Putative OTUs of natural *O.sinensis.* EMC represent the samples derived from external mycelial cortices, Soil represent the samples derived from soil microhabitat

Heatmap were performed based on the abundance information of each one from top 35 genera in different samples, which can visually reveal that the fungal communities in the external mycelial cortices of natural *O. sinensis* were significantly different from soil microhabitat samples (Fig. 3). The UniFrac-weighted Principal Coordinate Analysis (PCoA) showed that JM-1, JM-2, JM-3 were clustered together, TY-2, TY-3, TY-4 were separated on other side, which revealed that there are significant differences in fungal community structure between external mycelial cortices and soil microhabitat (Fig. 4). Dispersed state was observed among the samples from soil microhabitat collected from different sites, which indicating that the fungal communities composition were highly varied significantly in soil microhabitat across different areas. Venn diagram analysis revealed that 61 OTUs were exclusive to external mycelial cortices, 182 OTUs were exclusive to soil microhabitat, and 109 OTUs were shared (Fig. 5). Comparative analysis at the OTU level reveal that caterpillar fungus *O. sinensis* was overwhelmingly dominant in the external mycelial cortices, with proportion of 95.85%. Other predominant fungal species including *Archaeorhizomyces* sp., *Sebacina dimitica*, unclassified Ascomycota, accounted with 0.85, 0.86, 0.24%, respectively. However, unclassified *Inocybe*, *Archaeorhizomyces* sp., unclassified *Thelephoraceae*, unclassified *Tomentella*, *Thelephora* sp., unclassified Ascomycota, unclassified fungi, *Sebacina dimitica* and unclassified *Sebacina* were predominant fungal species in soil microhabitat, with proportion of 53.03, 8.69, 8.12, 8.09, 7.21, 3.08%, 3.05, 2.63, 1.57%, respectively (Fig. 6).

### Diversity of cultivable fungi

The surface sterilization effect meet the requirements of this study. Culture-dependent approach provide better taxonomic resolution than high-throughput sequencing. The fungal isolates varied with the different types of media, a total of 77 fungi were isolated from external mycelial cortices and soil microhabitat using culture-dependent approach. Firstly, fungal isolates were classified according to the characteristics of the colonies. The representative fungal isolates were identified by ITS region of the rDNA gene. All sequenced fungal isolates belong to 2 fungal phyla, 4 Classes, 9 orders, 21 families, 33 genera, Ascomycota and Zygomycota were predominant fungi, with proportion of 77.33, 22.67%, respectively. A total of 33 fungal genera, including *Ophiocordyceps*, *Trichoderma*, *Cytospora*, *Truncatella*, *Dactylonectria*, *Isaria*, *Cephalosporium*, *Fusarium*, *Cosmospora*, *Paecilomyces*, *Tolypocladium*, *Cercophora*, *Beauveria*, *Neonectria*, *Microdochium*, *Coniochaeta*, *Lecanicillium*, *Ilyonectria*, *Stagonosporopsis*, *Cladosporium*, *Pleotrichocladium*, *Leptosphaeria*, *Chaetosphaeronema*, *Paraphaeosphaeria*, *Preussia*, *Neosetophoma*, *Leptodontidium*, *Chalara*, *Pseudogymnoascus*, *Geomyces*, *Tetracladium*, *Mucor* and *Mortierella*, among of which, the predominant ones were *Mortierella* and *Trichoderma* (Table 3).

**Table 3:** Phylogenetic affiliations of cultivable fungi isolated from natural *O.sinensis*

Order	Isolates number	Closest identified relative	Accessionnumber	Identity (%)%
Ascomycota	TY105	*Valsa leucostoma*	KF294008	99
	TY110	*Leptosphaeria sclerotioides* strain IHBF 2251	MF326616	100
	TY112	*Truncatella angustata* strain SH9KPN	KT963797	100
	TY114	*Cladosporium* sp. CLJ-7	LC373150	100
	TY127	*Trichoderma polysporum* isolate CTCCSJ-F-ZY40741	KY750323	100
	TY128	*Trichoderma polysporum* isolate TR3.3	KX343127	100
	TY129	*Stagonosporopsis astragali* strain AS1S2–1	KP117286	100
	TY135	*Dactylonectria hordeicola* isolate EFA 443	MF440368	100
	TY137	*Trichocladium opacum*	HF678530	100
	TY138	*Isaria farinosa strain* IHBF 2244	MF326609	100
	TY139	*Chalara* sp. TMS-2011	HQ630988	100
	TY141	*Leptodontidium* sp. nc_besc_890f	HG936157	99
	TY147	*Preussia* sp. AU_CryP01	KC333159	99
	TY149	*Paraphaeosphaeria sporulosa* isolate F08–02	KX664338	99
	TY151	*Isaria fumosorosea* isolate FFJC 27	KF876832	99
	TY152	*Fusarium tricinctum* strain WBS031	KU350740	100
	TY154	*Cladosporium* sp. strain 2–3	KX378909	100
	TY156	*Paraphaeosphaeria* sp. QTYC56	KM103298	98
	TY162	*Cephalosporium* sp. PF1_NA_8	KT200264	97
	TY165	*Pseudogymnoascus* sp. isolate UFMGCB 10326	MG001401	100
	TY167	*Tetracladium* sp. P_S3	KP411581	99
	TY175	*Leptosphaeria* sp. QLF95	FJ025183	100
	TY182	*Paraphaeosphaeria* sp. QTYC50	KM103303	99
	TY185	*Cosmospora viridescens* IMI 73377a	NR_154791	95
	TY187	*Paecilomyces hepiali*	KX237743	99
	JM16	*Truncatella angustata* strain C23RB1	KT582088	100
	JM20	*Neonectria ramulariae* isolate F744	KM249079	99
	JM22	*Fusarium verticillioides* strain NSH-5	KX853851	100
	JM31	*Trichoderma paraviridescens* isolate CTCCSJ-G-JK40841	KY750503	100
	JM35	*Trichoderma polysporum* isolate CTCCSJ-G-HB40843	KY750506	99
	JM37	*Chaetosphaeronema achilleae* MFLUCC 16–0476	NR_153927	100
	JM42	*Tolypocladium cylindrosporum* strain IHBF 2265	MF326612	100
	JM45	*Neonectria candida*	MG000969	100
	JM46	*Ilyonectria sp.* strain P6011	KT270205	100
	JM48	*Neosetophoma* sp. strain P1802	KT269074	99
	JM51	*Neonectria candida* isolate VTN10Bs3	KU588183	99
	JM57	*Microdochium* sp. 5/97–31	AM502258	100
	JM60	*Trichoderma viridescens* voucher CTCCSJ-G-QT40323	MF928756	100
	JM63	*Fusarium tricinctum* isolate SBR01	KX823410	99
	JM64	*Geomyces* sp. AR-2009c	GU166479	100
	JM66	*Trichoderma nybergianum* CBS 122500	NR_134400	100
	JM74	*Leptosphaeria* sp. QLF95	FJ025183	97
	JM76	*Tetracladium* sp. P_S3_F	KP411581	99
	JM78	*Cercophora sulphurella* strain SMH2531	AY587913	97
	JM84	*Cladosporium cladosporiodies* isolate IGFRIWE10	MF171065	100
	JM100	*Trichoderma polysporum* isolate CTCCSJ-G-HB40843	KY750506.1	99
	JM112	*Hypocrea pachybasioides* strain T-50	KC884807	100
	JM114	*Beauveria bassiana* strain BLe-06	JX149538	100
	JM117	*Paraphaeosphaeria sporulosa* isolate F08–02	KX664338	99
	JM125	*Helotiales* sp. strain P2929	KT270126	99
	JM126	*Tolypocladium cylindrosporum*	AB208110	99
	JM129	*Helotiales* sp. MKOTU39	KP714632	100
	JM131	*Pseudogymnoascus* sp. APA-2015	KP902683	99
	JM132	*Cladosporium* sp. CLJ-7	LC373150	100
	JM137	*Paecilomyces farinosus* strain RCEF446	AF368797	99
	JM139	*Neonectria* sp. CJL-2014 strain Rc-R-30	KJ542219	100
	JM142	*Lecythophora* sp. NG_p46	HQ115712	99
	JM143	*Lecanicillium* sp. strain UFSMQ06	KX496884	100
	JM144	*Beauveria bassiana* strain BLe-06	JX149538	99
	ZG_1	*Ophiocordyceps sinensis* isolate 1229	KC184161	99
Zygomycota	TY100	*Mortierella minutissima* strain JZ-26	HQ637328	100
	TY101	*Mortierella* sp. QLF53	FJ025192	100
	TY103	*Mortierella* sp.	HG935763	99
	TY104	*Mortierella alpina* isolate QL-15	MF939657	100
	TY113	*Mortierella* sp. isolate UFMGCB 10336	MG001402	100
	TY117	*Mortierella antarctica* strain IHBF 2264	MF326600	100
	JM1	*Mucor hiemalis* isolate 349Jc14	KU516636	100
	JM11	*Mortierella alpina* isolate HG35	KU523253	99
	JM15	*Mortierella* sp. T_S4	KP411583	100
	JM25	*Mortierella hyalina* isolate FFJC 24	KF876828	97
	JM30	*Mortierella elongata* strain IHBF 2303	MF326586	99
	JM85	*Mortierella* sp. JZ-68	HQ637326	100
	JM91	*Mortierella* sp. GW20–2	JQ670951	99
	JM99	*Mortierella* sp. isolate N-4	MF939652	99
	JM105	*Mortierella alpina*	AB476411	99
	JM109	*Mortierella elongata* strain PFY	JX155654	100
	JM113	*Mortierella* sp. 02NH02	JX270348	99

TY indicates the fungi isolated from soil microhabitat, JM indicates the fungi isolated from external mycelial cortices

### Comparison of culture-dependent and-independent approaches

Surprisingly, there are significant differences in fungal communities of external mycelial cortices and soil microhabitat between two approaches. In external mycelial cortices, many ectomycorrhizal fungi, Orchidaceae and lichen symbiotic fungi were discovered by high-throughput sequencing, including, *Tetracladium maxilliforme*, *Pyronemataceae* sp., unclassified *Xylariales*, *Rachicladosporium antarcticum*, *Sebacinaceae*, unclassified *Sebacinales* and *Fusidium griseum*, etc.. However, several entomopathogenic fungi and *Trichoderma* sp. fungi were isolated using culture-dependent approach, including *Tolypocladium cylindrosporum*, *Beauveria bassiana*, *Lecanicillium* sp., *Paecilomyces farinosus*, *Trichoderma paraviridescens* and *Trichoderma viridescens*, etc.. In soil microhabitat, many cold adapted yeast, lichen symbiotic fungi, endophyte fungi and phytopathogenic fungi were discovered by high-throughput sequencing, including, *Cryptococcus terricola*, *Cryptococcus victoriae*, *Elasticomyces elasticus*, *Rhodotorula lamellibrachiae* and *Alatospora* sp., etc.. Using culture-dependent approach, we isolated many fungal species from soil microhabitat, which belong to *Mortierella* sp., entomopathogenic fungi and endophyte fungi, e.g., *Mortierella minutissima*, *Isaria farinosa*, *Isaria fumosorosea* and *Paraphaeosphaeria sporulosa*, etc..

## Discussion

The caterpillar fungus *O. sinensis* infects larvae of its hosts by oral cavity, insect cuticles or spiracle. After entering the hemolymph of host insect, the fungus grows as a hyphal body and threadlike hyphae, and converts it into caterpillar-shaped sclerotium. In the following year, mid-April to early June, the fruiting body bud growing on the head of sclerotium, emerging above the soil surface and forming a stalked fruiting body. Finally, ascospores were erupted from the mature fruiting body of *O. sinensis*, scatter in top soils, gradually infiltrate deeper into the soil, begin a new infection cycle. The dynamic changes and diversity of *O. sinensis* associated fungi in soil regulate the relationship of fungus-larvae, influence the occurence and geographical distribution of *O. sinensis* [27]. In Qinghai-Tibet Plateau, fungi community also are key components of the fragile ecosystems, perform critical ecological functions in biogeochemical cycles by decomposing organic matter and recycling nutrients. Uncovering fungi community composition of natural *O. sinensis* have great significance for better elucidating the interaction between microbiome and *O. sinensis*, the occurence and morphological variation of *O. sinensis.* However, it still lacked comprehensive investigation because of limitation of single approach.

In present study, 5 fungal phyla, 112 genera, and 352 OTUs were identifed using Illumina MiSeq sequencing, a total of 43 fungal genera were discovered in external mycelial cortices, *Ophiocordyceps*, *Sebacina* and *Archaeorhizomyces* were predominant genera accounted for 95.86, 1.14, 0.85%. A total of 66 fungal genera were discovered from soil microhabitat, *Inocybe*, *Archaeorhizomyces*, unclassified *Thelephoraceae*, *Tomentella*, *Thelephora*, *Sebacina*, unclassified *Ascomycota* and unclassified fungi were dominant genera with an average abundance of 53.32, 8.69, 8.12, 8.12, 7.21, 4.6, 3.08 and 3.05%, respectively. Previous study revealed that *Ophiocordyceps* was overwhelmingly dominant in the fruiting bodies and external mycelial cortices of the Chinese *Cordyceps*, which is consistent with our data. However, in soil samples, *Ophiocordyceps*, *Verticillium*, *Pseudallescheria*, *Candida*, *Ilyonectria*, *Neonectria* and *Fusarium* were the dominant genera [19]. Another previous study based on HiSeq sequencing of ITS genes of soil samples derived from the site that had a high density of *Thitarodes* larvae and Chinese *Cordyceps* at Shergyla Mountain revealed that *Archaeorhizomyces*, *Hyphodiscus*, *Beutheromyces*, *Pezoloma*, *Venturia*, *Geogiossum*, *Clavulinopsis*, *Cotylidia*, *Rammanopsis*, *Peltigera* and *Cavana* had high relative abundance [28]. Previous studies are inconsistent with each other, and also differ from our results, presumably, due to geographical origin, sampling processing and limitations of high-throughput sequencing, etc., which indicated that the *O. sinensis* associated microbial communities needs further study. In present study, 33 genera were isolated and identified using culture-dependent approach with different types of midia incubated at 16 °C, which mimic the true growth temperature of natural *O. sinensis. Mortierella* and *Trichoderma* were predominant culturable fungal genera. The data obtained from previous study showed that *Penicillium chrysogenum* is the predominant fungal species from fruiting body, and *Pseudogymnoascus roseus* is the dominant fungal species from sclerotia and external mycelial cortices using PDA incubated at 20 °C [8]. In our study, *Pseudogymnoascus* sp., was isolated from external mycelial cortices, but was not a predominant fungal species. There are significant differences in distinct studies, presumably, owing to different culture temperatures, media, sampling process and geographical origin, etc., which reflect the complexity of microbial communities from natural *O. sinensis.*

Compared with culture dependent approach which had limitations because of the selectivity of media and culture conditions, high-throughput sequencing was considered to provide better resolution of microbial community composition, but, it still showed biases. Each approach tend to capture different microbial community fractions, result in only a fraction of the taxa recovered did overlap, which also highlighting the complementarity of two approaches [29, 30]. Therefore, it is believed that the coordination of culture-dependent and independent approaches would provide promising avenue to illuminate microbial community assembly in natural *O. sinensis*. The idea was supported by our results obtained from two approaches. Using high-throughput sequencing, many ectomycorrhizal fungi, Orchidaceae and lichen symbiotic fungi were unexpectedly discovered in external mycelial cortices. Although rarefaction curves combined with the estimated coverage values indicated that overwhelming majority of fungal species were revealed from the natural *O. sinensis*. There were only 2 fungal species obtained from culture dependent approach were detected by high-throughput sequencing, i.e., *Tetracladium* sp. and *Lecythophora* sp.. Significantly, several entomopathogenic fungi and *Trichoderma* sp. were isolated frequently using culture-dependent approach.

Various environmental factors, including terrain, vegetation characteristics, altitude, climate change and anthropogenic disturbance, etc., strongly regulate soil properties, soil temperature and moisture, which alter the microclimate, then shape microbial communities composition of soil microhabitat [31]. The alpine meadow ecosystem of the Qinghai-Tibetan Plateau that suitable for the occurrence of *O. sinensis*, are the most fragile and sensitive to environmental factors change. The dynamic alterations of microbial community in natural habitat *O. sinensis* response to seasonal temperature fluctuation, are also need further study. Furthermore, soil microbial communities strongly influence the yield and occurrence of natural *O. sinensis*, because of the infection of host *Thitarodes* larvae mainly occurs in soils [28]. The suitable habitat of *O. sinensis* and host insects mainly contain *Polygonum viviparum*, *Polygonum macrophyllum* D.Don., *Polygonum* s*phaerostachyum* Meisn., *Polygonatum curvistylum* and *Rheum tibeticum* Maxim. ex Hook.f., etc. [32]. The data from high-throughput sequencing reveal that ectomycorrhizal fungi, Orchidaceae and lichen symbiotic fungi, plant pathogenic fungi, endophytic fungi and large number of novel fungal species were widespread in soil microhabitat of natural *O. sinensis*. The fungus *O. sinensis* occupy the vast majority of fungal community in external mycelial cortices, has a selective effect on symbiotic and associated microbial species, as there are still many plant endophytic fungi in microbial community.

Regarding entomopathogenic fungi in natural *O. sinensis*, *Tolypocladium cylindrosporum*, *Paecilomyces farinosus*, *Isaria farinosa*, *Isaria fumosorose*, *Lecanicillium sp*. and *Beauveria bassiana* were isolated using culture-dependent approach in our results. Previous study revealed that *Metarhizium*, *Pochonia*, *Simplicillium*, *Elaphocordyceps*, *Polycephalomyces*, *Purpureocillium* and *Tolypocladium* are present in the Chinese *Cordyceps* [28]. *Sebacina*, a mycorrhizal fungus frequently isolated from Orchidaceae and plant, promote growth, enhance stress resistance and seed germination, was in high abundance in external mycelial cortices [33]. *Archaeorhizomyces*, a widespread fungal class with a dominant presence in many soil environments, increase plant bioactive components, enhance stress resistance and inhibit the occurrence of plant diseases by different mechanisms, also was high relative abundance genus in external mycelial cortices. *Trichoderma* spp. are proposed as major plant growth-promoting fungi in soils, significantly alter the rhizosphere soil chemistry, regulate microbial communities and improve grassland biomass [34]. *Trichoderma* species were frequently isolated from external mycelial cortices by culture-dependent approach, and also have been identified by high-throughput sequencing. Accumulated evidence and our data support the hypothesis that the caterpillar fungus *O. sinensis* parasitize the larvae of *Thitarodes* to form larva shape sclerotia, produce diverse secondary metabolites or organic ingredients, which could alter soil characteristic surrounding *O. sinensis*, influence the microbial community composition, form a complex host-microbiota microecosystem. The total protein content, pharmacological effect and bioactive components of natural *O. sinensis* are much higher than artificially cultivated *O. sinensis*, which indicate that shaping symbiotic and associated microbial species are significantly important in the growth and development of *O. sinensis* and formation of perithecial fruiting bodies. Presumably, these associated fungi might be the real producer of certain pharmacologically bioactive components in natural *O. sinensis*. A case of Truffles (*Tuber* spp.), are well known for their enticing aromas partially emitted by microbes colonizing truffle fruiting bodies, their associated microbes related to stimulation of the growth of Tuber mycelium, inhibition of pathogenic fungi, the elaboration of the complex aroma of truffles [35]. The data presented here highlight the importance of several fungi in *O. sinensis*, namely *Trichoderma* sp., *Archaeorhizomyces*, *Sebacina*, insect pathogenic fungi and mycorrhizal fungi, etc., the synergistic effect of different fungi help to produce *O. sinensis*.

Some studies demonstrate that other microbial species could colonize inside sclerotia except the caterpillar fungus *O. sinensis. Cordyceps* related fungal species, namely, *P. hepiali*, *T. sinensis*, *P. sinensis*, *Synnematium sinense* and C*ephalosporium dongchongxiacae*, etc., most of which belong to entomopathogenic fungi, were isolated from fruiting body or sclerotia, which resulting in the debate of unique anamorph of *O. sinensis* lasted for decades. Here, aiming to explain this fact, based on our data and previous studies, we hypothesized that the larvae of *Thitarodes* sp., were infected by *O. sinensis* accompanied with degradation of insect cuticle and tissue, which drive the soil microhabitat around *O. sinensis* highly suitable for entomopathogenic fungi, improve the abundance of various types of entomopathogenic fungi and parasitize inside the sclerotia. Yet, there are significant variations of entomopathogenic fungi species in microbial community of *O. sinensis* derived from different geographical region. The present study, taking internal tissue from sterilized fruiting body and sclerotia as material, only the fungus *O. sinensis* was isolated using traditional cultivated approach. However, 5 fungal species were isolated from the sterilized sclerotia surface tissue. This result is consistent with some previous finding that total 2 unique OTUs were isolated from fruiting body, 4 OTUs from sclerotia, and 115 OTUs from mycelial cortices [9]. However, some previous data revealed that many microbial species identified from fruiting body and sclerotia, presumably, due to the fact that the soil attached to sample were not removed. Our research aimed to reveal the fungal diversity in microecosystem of *O. sinensis*, and to elucidate the role of fungal community composition in the occurrence of *O. sinensis*. Therefore, we selected the external mycelial cortices and soil microhabitat for study using coordination of culture-dependent and independent approaches.

The soil microhabitat and *O. sinensis* form a complex microecosystem, which harbour huge number of fungi, bacteria and actinomycetes. The interactions among soil microbial community might play a significant role in the infection and occurrence of *O. sinensis*. Bacterial diversity of natural *O. sinensis* was also investigated by high-throughput sequencing in our group. In external mycelial cortices, 1667 bacterial OTUs, belonging to 17 genera were identified, the predominant genera including others (48.92%), norank *Acidobacteria* (5.39%), RB41 (4.16%), *Flavobacterium* (3.15%), norank *Nitrosomonadaceae* (2.81%), *Bradyrhizobium* (2.66%), *Nitrospira* (2.42%) and norank *Anaerolineaceae* (2.01%*)*, etc.. In soil microhabitat, 1676 bacterial OTUs, belonging to 18 genera also were identified, and 1623 bacterial OTUs were shared (unpublished data). The average relative abundance of several bacterial genera, i.e., *Flavobacterium*, *Ferruginibacter* and *Pseudomonas* were higher in external mycelial cortices, indicated that these bacterial species might enhance fungal mycelial growth and promoting the occurence of *O. sinensis*. Recent evidence revealed that *Pseudomonas* sp. P7014 can enhance mycelial growth and reduce harvesting time of *Pleurotus eryngii* [36]. However, no studies have yet reported the best growth promoting microbes for *O. sinensis*, and it would be a hot topics.

*O. sinensis* and its soil microhabitat represent the potential source to explore microbial biosynthetic diversity and evolutionarily ancient examples of symbiosis, for some of *O. sinensis* associated fungi produce similar bioactive metabolites [6, 37, 38]. In microecosystem, polyketide synthase and nonribosomal peptide synthetase, the large multidomain and multifunctional megaenzymes involved in biosynthesis of secondary metabolites, evolve rapidly through horizontal transfer from bacteria to fungi, particularly, between fungi, which contribute to produce vast numbers of novel metabolites [39]. Presumably, horizontal gene transfer frequently occur in the microbiota of natural *O. sinensis* to improve the genomic flexibility and produce structurally similar metabolites during long-term symbiosis and evolution.

Previous study confirmed that the host insects of *O. sinensis* from different geographic origin are complex, mostly in the genus *Hepialus* and to a lesser extent in the genera *Hepialiscus*, *Forkalus* and *Bipectilus* [40]. Furthermore, the fungus *O. sinensis* and its hosts have coevolved. To better clarify the structure of microbial community of *O. sinensis*, extensively collecting sampling across the geographical distribution of *O. sinensis* are necessary. However, sampling collection was affected by traffic, sample storage and shorter period of emergence of *O. sinensis.* Here, we aim to unravel fungal communities of natural *O. sinensis* using combination of high-throughput sequencing and culture-dependent approach. Therefore, *O. sinensis* samples were collected at five sites from Golog Tibetan Autonomous Prefecture of Qinghai Province, which is a suitable geographical distribution of *O. sinensis* with high quality and yield. Although the limited sampling sites covered by this study possibly result in decreasing of credibility of the present results, considerable previous studies support our data and hypothesis [19, 41].

As well know, *O. sinensis* usually occur in the humus-rich horizon of soils. Unfortunately, in present study, we did not determine the characteristics of the soil samples. Other study reveal that the pH values of habitat soils of natural *O. sinensis* range from 5.01 to 6.00, and no significant difference among different habitats. The soil microhabitat of *O. sinensis* contain a large amount of organic matters and nitrogen, which control the whole microbial community composition and affect the occurence of *O. sinensis.* Further studies are needed to confirm the relationship between soil properties and microbial community composition and the occurence of *O. sinensis.* In present study, we only divided the sample into external mycelial cortices and soil microhabitat. The dynamics of the hyphal bodies and it’s density in different distance from *O. sinensis* larvae in soil microhabitat are worthing further study, which would providing novel insight to occurrence mechanism of *O. sinensis* and be benefit to overcome the obstacle in artificial culture of *O. sinensis.*

## Conclusion

This study revealed that each approach tend to capture different microbial community fractions, the significantly differences and little overlap in fungal community of natural *O. sinensis* between two approaches highlight that the combination of high-throughput sequencing and culture-dependent approaches would generate more information. A comprehensive investigation of the fungal community structure in natural *O. sinensis* performed by two approaches indicated that microbial species, namely *Trichoderma* sp., *Archaeorhizomyces*, *Sebacina*, entomogenous fungi and some bacterial species, etc., play an important role in the occurence and maturation of *O. sinensis*. In a word, our result provide novel insights into the fungal community structure of natural *O. sinensis*, reveal the occurrence mechanism of *O. sinensis*, and would be beneficial to overcome the obstacle in artificial culture of *O. sinensis.*

## Methods

### Site description, sampling collection and processing

The size and shape of *O. sinensis* vary in different areas owing to the variety of infected *Thitarodes* insects, environmental factors, including plant species, soil characteristic and soil microbial community, etc.. Therefore, these *O. sinensis* samples were collected at five sites from shrubland and alpine meadow with altitude of 4000–5200 m, which distributes in the territory of Maqen County, Darlag County and Gade County, Golog Tibetan Autonomous Prefecture of Qinghai Province (97°54′-101°50’ longitude, 32°31′-35°40′ latitude) during May 10–20, 2015. These regions are representative geographical distribution of the *O. sinensis* with high quality and density. Five repeats were performed by the five-point method at each site. During sampling, the naturally growing *O. sinensis* was taken as the center point, soil block with 15 cm diameter and depth of 20 cm were collected. The samples were transported to the laboratory within 24 h under controlled temperature (4 °C). Processed samples were stored at 4°Cfor fungal isolation and at − 80°Cfor DNA extraction, respectively.

**Fig. 7:**
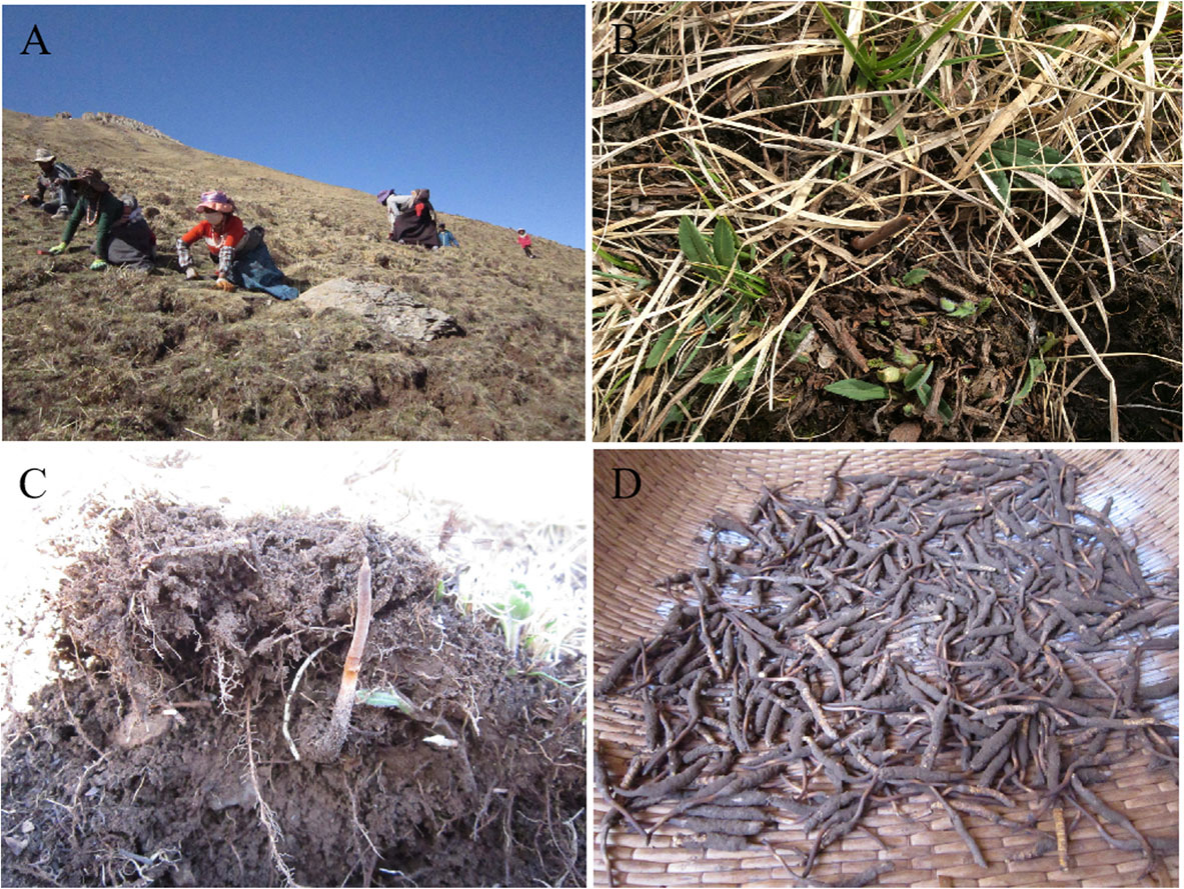
Natural habitat and collection of *O. sinensis*s samples. **a** Natural habitat for collecting *O. sinensis* samples; **b** Growing *O. sinensis*; **c** Soil microhabitat surrounding natural *O. sinensis*; **d** External mycelial cortices including the mycelial cortices covering *O. sinensis* larvae and soil particles naturally attached to the surface of larvae

Firstly, plant residues and stones in samples were removed with a sieve. The samples derived from same site were divided into three subsamples. (i) External mycelial cortices including the mycelial cortices covering *O. sinensis* larvae and soil particles naturally attached to the surface of larvae. The names of the three repeat samples from different sites were designated as “JM-1, JM-2, JM-3”. (ii) Soil microhabitat, represent a specific areas of soil surrounding natural *O. sinensis*, in which *O. sinensis* interact with soil microbial community. The names of the three repeat samples from different sites were abbreviated as “TY-2, TY-3, TY-4”. (iii) Fruiting body of *O. sinensis* represent the part that infected fungus growing on the head of caterpillar-shaped sclerotium. Samples from external mycelial cortices and soil microhabitat were investigated using high-throughput sequencing and culture-dependent approaches, yet, fruiting body was investigated by culture-dependent approach because of little microbial species (Fig. 7).

### Culture media used for fungal isolation

For best efforts to isolate fungal species from external mycelial cortices, soil microhabitat and fruiting body of natural *O. sinensis*, seven types of media were used, namely, potato dextrose agar (PDA) medium (extract from 200 g boiled potatoes, 20 g glucose, 20 g agar, 800 mL distilled water, natural pH), 1/4 PDA medium (extract from 50 g boiled potatoes, 5 g glucose, 2.5 g peptone, 15 g agar, 1000 mL distilled water, natural pH), modified PDA (I) (PDA medium+ 1% peptone+ 1% yeast extract), modified PDA (II) [PDA medium+ 1 g KH_2_PO_4_, 1.5 g MgSO_4_.7H_2_O, 0.2 g MgCl_2_, 0.2 g (NH_4_)_3_PO_4_], nutrient rich medium (10 g glucose, 10 g peptone, 10 g silkworm pupal powder, 1 g KH_2_PO_4_, 0.5 g MgSO_4_.7H_2_O, 20 g agar, 1000 mL of distilled water, pH 6.8), G medium (extract from 200 g boiled potatoes, 10 g peptone, 20 g silkworm pupae powder, vitamin B_1_, 15 agar, 1000 mL distilled water, PPDA medium (PDA medium+ 1% peptone).

### DNA extraction, high-throughput sequencing

For fungal community description, total genomic DNA was extracted from each subsamples, i.e., external mycelial cortices and soil microhabitat using two approaches, respectively. To improve the efficiency of the microbial DNA extraction, the samples were crushed after freezing with liquid nitrogen in precooled mortar. The total genomic DNA extracted by two approaches were pooled for further step.

First approach. Total genomic DNA extraction from external mycelial cortices and soil microhabitat were performed using the MoBio PowerSoil DNA Isolation Kit (MoBio Laboratories, Carlsbad, CA, USA) according to manufacturer’s instructions, respectively. The integrity of genomic DNA was checked by 1.0% agarose gel electrophoresis (1% w/v).

Secondary approach. Before DNA extraction, the samples were treated using TENP buffer (50 mM Tris, 20 mM EDTA, 100 mM NaCl, 0.01 g/ml Polyvinylpyrrolidone, pH 10) and PBS buffer (137 mmol/L NaCl, 2.7 mmol/L KCl, 10 mmol/L Na_2_HPO_4_, 2 mmol/L KH_2_PO_4_, pH 7.4) to remove humus. Then, the genomic DNA was extracted using the traditional method with minor modification. Briefly, For each 2 g samples, add 3 mL DNA extraction buffer, 20 μL proteinase K, 30 μL lysozyme and 20 μL lytic enzyme, mix by vortexing 30 min, add 0.5 mL 20% SDS, subsequently incubated for 2 h at 65 °C. Solution was centrifuged at room temperature 9000 r/min for 15 min, the supernatant was transferred to a new centrifuge tube. Equal volume of saturated phenol: chloroform: isoamyl alcohol (25,24,1) were used to remove protein contamination, and isopropanol was used to precipitate DNA for 30 min, followed by centrifugation at 6000×g for 30 min. Supernatant was discarded, pellet was washed with 70% ethanol, centrifuged again. Subsequently, the supernatant was removed, air dried and finally resuspended in an appropriate amount of Tris-EDTA buffer (10 mM Tris, 1 mM EDTA, pH 8.0).

The concentration and purification of mixed genomic DNA extracted using the above two approaches were determined by NanoDrop 2000 UV-vis spectrophotometer (Thermo Scientific, Wilmington, USA), the fungal rDNA ITS1 regions were amplified from the mixed total genomic DNA with primers ITSIF (5′-CTTGGTCATTTAGAGGAAGTAA-3′) and ITS2R (5′-GCTGCGTTCTTCATCGATGC-3′) by the ABI GeneAmp® 9700 PCR System (Applied Biosystems, Waltham, MA, USA) [42]. The PCR reaction mixture (20 μL) contained 4 μL of 5× FastPfu Buffer, 2 μL of dNTPs (2.5 mM), 0.8 μL of each primer, 0.4 μL of FastPfu Polymerase, 10 ng of template DNA. The PCR procedures were 95 °C for 3 min initial denaturation, 27 cycles of 30 s at 95 °C, 30 s for annealing at 55 °C, 45 s for elongation at 72 °C, and a final extension at 72 °C for 10 min. Triplicate PCR reactions were carried out for each sample, the products were mixed, and evaluated by 2% agarose gel electrophoresis. The mixed products were purified using the AxyPrep DNA Gel Extraction Kit (Axygen Biosciences, Union City, CA, USA) and quantified using QuantiFluor™-ST (Promega, USA). Purified amplicons were pooled in equimolar and paired-end sequenced (2 × 300) on an Illumina MiSeq platform (Illumina, San Diego, USA) according to the standard protocols by Majorbio Bio-Pharm Technology Co. Ltd. (Shanghai, China). Raw sequencing data obtained from this study were deposited at DDBJ/ENA/GenBank under the accession numbers: PRJNA609607.

### Processing of sequencing data

Raw FASTQ files were demultiplexed, quality-filtered by Trimmomatic, and merged by the following criteria: (i) The reads were truncated at any site receiving an average quality score < 20 over a 50 bp sliding window. (ii) Primers were exactly matched allowing 2 nucleotide mismatching, and reads containing ambiguous bases were removed. (iii) Sequences whose overlap longer than 10 bp were merged according to their overlap sequence. After quality control, the reads were clustered into OTUs according to the pipeline of QIIME commands based on 97% sequence similarity. Representative sequences of each OTU were blasted against the UNITE database to obtain taxonomy with a threshold value of 0.8. The α-diversity indices, including ACE index, Chao1 index, Shannon index, Simpson index, and Coverage were calculated using Mothur 1.30.1. Euclidean-based Weighted UniFrac distances were employed to determine the distance (β-diversity) between fungal communities in any pair of samples. Venn diagrams were generated to illustrate the proportion of shared and unique taxa between different samples. The hierarchical clustering graph was generated using the MeV software, version 4.9.0 and the hierarchical clustering method. Principal coordinates analysis (PCoA) is a visualization method for studying variation in data, based on UniFrac metrics, and is useful for describing the species composition similarity between samples. In present study, PCoA was performed using the vegan package 2.0–10. All software used was in Mothur package.

### Isolation and identification of culturable fungi

The culturable fungi were isolated from the external mycelial cortices and soil microhabitat, which were pooled from all samples derived from different sites by the dilution-plate approach. A total of 10 g samples were added to a triangular flask containing 90 mL of sterile water and glass beads, shaking at 180 r/min for 1 h at 16 °C, to prepare a suspension with a concentration of 10^− 1^, then serially diluted with sterile water to a concentration of 10^− 2^–10^− 6^. A total of 200 μL of each dilution were plated with triplicates in seven types of media. All of which contain 100 μg/mL streptomycin, 100 μg/mL penicillin and 10 mg/L rose bengal. The Petri dishes were incubated at 16 °C in dark, and the characteristic of each emerging fungal colony was observed daily, then transferred onto a new PDA plate, until axenic cultures were obtained.

Fruiting body and sclerotia of *O. sinensis* were rinsed with sterile water, surface-sterilized using 75% ethanol for 3 min, followed by 2.5% sodium hypochlorite (NaClO) for 25 min, rinsed twice in sterile distilled water, dried on sterile filter paper. Subsequently, the internal tissue blocks obtained from the fruiting body and sclerotia of sterilized *O. sinensis*, were cut into 1–2 mm slice, then placed on different media described above. The Petri dishes were incubated at 16 °C in dark. While hyphae germinate and grow from the tissue slice, the tip of hyphae were picked to purify culturable fungal species.

Effectiveness of surface sterilization: the surface-sterilized samples were washed in sterile distilled water three times, soaked in 1.5 mL sterile water, and stirred for 1 min. A total of 100 μL suspension were inoculated onto modified PDA (I) medium plates at 16 °C in dark. If no microbial growth occurred on the medium, the sterilization was considered complete.

### Identification of culturable fungal isolates

The purified fungal isolates were categorized preliminarily based on colony characteristics, including colony morphology, texture, aerial hyphae, substrate hyphae, spore mass color, sporophore and spore chain morphology, distinctive reverse colony color and diffusible pigment, etc.. The representative fungal isolates with distinct morphology were identified using a combination of morphology characteristics and the ITS sequence. The extraction of genomic DNA were performed using CTAB methods as previously described [43].

The nearly full-length ITS region of the rDNA gene was amplified by the primers ITS1 (5′-TCCGTAGGTGAACCTGCGG-3′) and ITS4 (5′-TCCTCCGCTTATTGATATGC-3′) [44]. The amplicons were purified using the ExoSAP-IT reagent (USB Corporation, Cleveland, OH) following manufacturer’s instructions, ligated into pMD18-T (TaKaRa, Japan) and transformed into competent *E.coli* DH5a following the manufacturer’s protocol. The positive transformants were sent to Shanghai Invitrogen Biotechnology Co. Ltd., for sequencing. The sequences of fungal rDNA ITS region were compared with that of the most closely-related fungal species (higher than 97% similarity) in the NCBI database using the BLAST program. The ITS sequence data were submitted and deposited in GenBank under accession numbers MT133906-MT134006.

## Data Availability

The datasets used and analysed during the current study are available from the corresponding author on reasonable request. The raw reads of MiSeq data were also deposited into the NCBI Sequence Read Archive database under accession numbers: PRJNA609607. All the ITS sequence data of 77 culturable fungal isolates have been upload to NCBI database under accession numbers: MT133906-MT134006.

## Abbreviations

OTUs: operational taxonomic units
EMC: external mycelial cortices
Soil: soil microhabitat
PCR: Polymerase Chain Reaction
PDA: potato dextrose agar
DNA: DeoxyriboNucleic Acid
SDS: Sodium dodecyl sulfate
ITS: internal transcribed spacer

## References

[1] Buenz EJ, Bauer BA, Osmundson TW, Motley TJ (2005). The traditional Chinese medicine *Cordyceps sinensis* and its effects on apoptotic homeostasis. J Ethnopharmacol.

[2] Li Y, Wang XL, Jiao L, Jiang Y, Li H, Jiang SP (2011). A survey of the geographic distribution of *Ophiocordyceps sinensis*. J Microbiol.

[3] Wu TR, Lin CS, Chang CJ, Lin TL, Martel J, Ko YF (2019). Gut commensal *Parabacteroides goldsteinii* plays a predominant role in the anti-obesity effects of polysaccharides isolated from *Hirsutella sinensis*. Gut..

[4] Jiraungkoorskul K, Jiraungkoorskul W. Review of naturopathy of medical mushroom, *Ophiocordyceps sinensis*, in sexual dysfunction. Pharmacogn Rev. 2016;10(19):1–5; doi: 10.4103/0973-7847.176566. 10.4103/0973-7847.176566 PMC4791983 27041868

[5] Zhang Y, Zhang S, Li Y, Ma S, Wang C, Xiang M (2014). Phylogeography and evolution of a fungal-insect association on the Tibetan plateau. Mol Ecol.

[6] Zhou X, Gong Z, Su Y, Lin J, Tang K (2009). *Cordyceps* fungi: natural products, pharmacological functions and developmental products. J Pharm Pharmacol.

[7] Guo LX, Xu XM, Liang FR, Yuan JP, Peng J, Wu CF (2015). Morphological observations and fatty acid composition of indoor-cultivated *Cordyceps sinensis* at a high-altitude laboratory on Sejila mountain. Tibet PLoS One.

[8] Zhang YJSB, Zhang S, Wang M, Liu XZ, Gong WF (2010). Mycobiotal investigation of natural *Ophiocordyceps sinensis* based on culture-dependent investigation. OALib Journal.

[9] Zhang Y, Zhang S, Wang M, Bai F, Liu X (2010). High diversity of the fungal community structure in naturally-occurring *Ophiocordyceps sinensis*. PLoS One.

[10] Choi HY, Stewart GM, Lomas MW, Kelly RP, Moran SB (2014). Linking the distribution of (210) Po and (210) Pb with plankton community along line P, northeast subarctic Pacific. J Environ Radioact.

[11] Chen YQ, Hu B, Xu F, Zhang W, Zhou H, Qu LH (2004). Genetic variation of *Cordyceps sinensis*, a fruit-body-producing entomopathogenic species from different geographical regions in China. FEMS Microbiol Lett.

[12] Shrestha UBBK (2013). Trade, harvest, and conservation of caterpillar fungus (*Ophiocordyceps sinensis*) in the Himalayas. Biol Conserv.

[13] Guo H, Sun B, Gao H, Chen X, Liu S, Yao X (2009). Diketopiperazines from the *Cordyceps*-colonizing fungus *Epicoccum nigrum*. J Nat Prod.

[14] Chen Y, Guo H, Du Z, Liu XZ, Che Y, Ye X (2009). Ecology-based screen identifies new metabolites from a *Cordyceps*-colonizing fungus as cancer cell proliferation inhibitors and apoptosis inducers. Cell Prolif.

[15] Wang J, Teng L, Liu Y, Hu W, Chen W, Hu X (2016). Studies on the antidiabetic and antinephritic activities of *Paecilomyces hepiali* water extract in diet-streptozotocin-induced diabetic Sprague dawley rats. J Diabetes Res.

[16] Li SP, Li P, Dong TT, Tsim KW (2001). Anti-oxidation activity of different types of natural *Cordyceps sinensis* and cultured *Cordyceps* mycelia. Phytomedicine..

[17] Rappe MS, Giovannoni SJ (2003). The uncultured microbial majority. Annu Rev Microbiol.

[18] Magnuson JK, Lasure LL (2002). Fungal diversity in soils as assessed by direct culture and molecular techniques.

[19] Xia F, Chen X, Guo MY, Bai XH, Liu Y, Shen GR (2016). High-throughput sequencing-based analysis of endogenetic fungal communities inhabiting the Chinese *Cordyceps* reveals unexpectedly high fungal diversity. Sci Rep.

[20] Melcher U, Verma R, Schneider WL (2014). Metagenomic search strategies for interactions among plants and multiple microbes. Front Plant Sci.

[21] Uroz S, Ioannidis P, Lengelle J, Cebron A, Morin E, Buee M (2013). Functional assays and metagenomic analyses reveals differences between the microbial communities inhabiting the soil horizons of a Norway spruce plantation. PLoS One.

[22] Becher D, Bernhardt J, Fuchs S, Riedel K (2013). Metaproteomics to unravel major microbial players in leaf litter and soil environments: challenges and perspectives. Proteomics..

[23] Loman NJ, Misra R (2012). V., Dallman, T. J., Constantinidou, C., Gharbia, S. E., Wain, J. et al. performance comparison of bench-top high-throughput sequencing platforms. Nat Biotechnol.

[24] Massana R (2011). Eukaryotic picoplankton in surface oceans. Annu Rev Microbiol.

[25] Oberauner L, Zachow C, Lackner S, Hogenauer C, Smolle KH, Berg G (2013). The ignored diversity: complex bacterial communities in intensive care units revealed by 16S pyrosequencing. Sci Rep.

[26] Liang Y, Hong Y, Mai Z, Zhu Q, Guo L. Internal and external microbial community of the *Thitarodes* moth, the host of *Ophiocordyceps sinensis*. Microorganisms. 2019;7(11); doi: 10.3390/microorganisms7110517. 10.3390/microorganisms7110517 PMC6920881 31683719

[27] Christensen M (1989). A view of fungal ecology. Mycologia..

[28] Shao JL, Lai B, Jiang W, Wang JT, Hong YH, Chen FB, et al. Diversity and co-occurrence patterns of soil bacterial and fungal communities of Chinese *Cordyceps* habitats at Shergyla mountain, Tibet: Implications for the Occurrence. Microorganisms. 2019;7(9); doi: 10.3390/microorganisms7090284. 10.3390/microorganisms7090284 PMC6780579 31443515

[29] Rego A, Raio F, Martins TP, Ribeiro H, Sousa AGG, Seneca J (2019). Actinobacteria and cyanobacteria diversity in terrestrial antarctic microenvironments evaluated by culture-dependent and independent methods. Front Microbiol.

[30] Li AZ, Han XB, Zhang MX, Zhou Y, Chen M, Yao Q (2019). Culture-dependent and-independent analyses reveal the diversity, structure, and assembly mechanism of benthic bacterial community in the Ross Sea. Antarctica Frontiers in microbiology.

[31] Zhang Y, Dong S, Gao Q, Liu S, Zhou H, Ganjurjav H (2016). Climate change and human activities altered the diversity and composition of soil microbial community in alpine grasslands of the Qinghai-Tibetan plateau. Sci Total Environ.

[32] Lei W, Zhang G, Peng Q, Liu X (2015). Development of *Ophiocordyceps sinensis* through plant-mediated interkingdom host colonization. Int J Mol Sci.

[33] Ray P, Guo Y, Kolape J, Craven KD (2017). Non-targeted colonization by the endomycorrhizal fungus, *Serendipita vermifera,* in three weeds typically co-occurring with switchgrass. Front Plant Sci.

[34] Zhang F, Huo Y, Cobb AB, Luo G, Zhou J, Yang G (2018). *Trichoderma* biofertilizer links to altered soil chemistry, altered microbial communities, and improved grassland biomass. Front Microbiol.

[35] Splivallo R, Vahdatzadeh M, Macia-Vicente JG, Molinier V, Peter M, Egli S (2019). Orchard conditions and fruiting body characteristics drive the microbiome of the black truffle *Tuber aestivum*. Front Microbiol.

[36] Kim MK, Math RK, Cho KM, Shin KJ, Kim JO, Ryu JS (2008). Effect of *Pseudomonas* sp. P7014 on the growth of edible mushroom *Pleurotus eryngii* in bottle culture for commercial production. Bioresour Technol.

[37] Ko YF, Liau JC, Lee CS, Chiu CY, Martel J, Lin CS (2017). Isolation, culture and characterization of *Hirsutella sinensis* mycelium from Caterpillar fungus fruiting body. PLoS One.

[38] Yang JL, Xiao W, He HX, Zhu HX, Wang SF, Cheng KD (2008). Molecular phylogenetic analysis of *Paecilomyces hepiali* and *Cordyceps sinensis*. Yao Xue Xue Bao.

[39] Fischbach MAWC, Clardy J (2008). The evolution of gene collectives: how natural selection drives chemical innovation. Proc Natl Acad Sci U S A.

[40] Quan QM, Chen LL, Wang X, Li S, Yang XL, Zhu YG (2014). Genetic diversity and distribution patterns of host insects of Caterpillar fungus *Ophiocordyceps sinensis* in the Qinghai-Tibet plateau. PLoS One.

[41] Xia F, Zhou X, Liu Y, Li Y, Bai X, Zhou X (2019). Composition and predictive functional analysis of bacterial communities inhabiting Chinese *Cordyceps* insight into conserved core microbiome. BMC Microbiol.

[42] Adams RI, Miletto M, Taylor JW, Bruns TD (2013). Dispersal in microbes: fungi in indoor air are dominated by outdoor air and show dispersal limitation at short distances. ISME J.

[43] Heinig USS, Jennewein S (2013). Getting to the bottom of Taxol biosynthesis by fungi. Fungal Divers.

[44] Rosa LH, Vaz ABM, Caligiorne RB, Campolina S, Rosa CA. Endophytic fungi associated with the Antarctic grass *Deschampsia antarctica* Desv. (Poaceae). Polar biology. 2008;32(2):161-7; doi: 10.1007/s00300-008-0515-z. QIIME (http://qiime.org/genindex.html). Venn diagrams. http://jura.wi.mit.edu/bioc/tools/venn3way/index.php.

